# Effect of alumina shapes on dielectric properties of UV-cured epoxy acrylic composite with alumina

**DOI:** 10.1098/rsos.181509

**Published:** 2019-01-16

**Authors:** Jiepeng Bian, Qiuli Zhao, Zhenzhong Hou, Jie Dong, Qinghao Yang, Guanjun Zhang

**Affiliations:** 1 College of Materials Science and Engineering, Xi'an University of Science and Technology, Xi'an 710054, People's Republic of China; 2 State Key Laboratory of Electrical Insulation and Power Equipment, School of Electrical Engineering, Xi'an Jiaotong University, Xi'an 710049, People's Republic of China

**Keywords:** composites, alumina shapes, dielectric properties, UV-cured epoxy acrylic

## Abstract

Polymer-based composites with the advantages of ceramics and polymers exhibit high dielectric constant, good processing properties and low dielectric loss. The composites with a varied content of irregular alumina (*i*-Al_2_O_3_) filler were prepared by UV-cured epoxy acrylic (EA). Spherical alumina (*s*-Al_2_O_3_) was used as a filler to further investigate the effect of alumina (Al_2_O_3_) shapes on dielectric properties of composites in the frequency range of 50 Hz–1 MHz. Fourier transform infrared spectroscopy proved that the UV-cured epoxy acrylic/alumina (Al_2_O_3_/EA) composites were successfully fabricated. Scanning electron microscopy demonstrated that *i*-Al_2_O_3_ particles have superior homodispersion in the matrix. Through testing, for all samples, with the addition of Al_2_O_3_, the relative permittivity of composites increased as expected, and the dielectric loss decreased accordingly. These data show that the incorporation of *i*-Al_2_O_3_ particles presents better properties when compared with *s*-Al_2_O_3_/EA, which indicates that *i*-Al_2_O_3_ particles have more influence on the dielectric properties of the composites than those of *s*-Al_2_O_3_ particles. According to Weibull distribution, the characteristic breakdown strength of the Al_2_O_3_/EA composites was obtained and the results suggested that the composites of *i*-Al_2_O_3_/EA exhibited better breakdown performance.

## 1. Introduction

Polymer-based insulation materials with high dielectric constant have been widely applied in the electrical and electronic field, especially in capacitors, motors and cables [1–3]. However, polymer materials generally exhibit low dielectric constant, and the energy density cannot be greatly improved [4], which limits the application of polymer materials in insulation field. Generally, one approach to enhance the relative permittivity of polymer is to incorporate the particles with a higher level of permittivity as fillers, such as inorganic ceramic powders (e.g. BaTiO_3_ and Ba*_x_*Sr_1−*x*_TiO_3_) [5–7] and particles with high electric conductivity like metal and carbon nanotubes [8–10]. Recently, Ghosh *et al*. [11] reported a composite film with polyvinylidene fluoride (PVDF) as matrix, and the silver (Ag) nanoparticles decorated CaCu_3_Ti_4_O_12_ (CCTO) as a filler showed high dielectric constant, which is about 20% higher than that of pure CCTO. He *et al*. [12] prepared a core–shell structure nanoparticle and used it as a filler to fabricate BT@Al_2_O_3_/PVDF composite films, which show a low dielectric loss (about 0.02 in 1 kHz) with high dielectric constant. Ceramic powders, metal particles and carbon nanotubes are incorporated in the polymer in an appropriate proportion, which can bring the advantages of both in composite materials [13–16]. Therefore, the preparation of polymer-based dielectric composites has become a hotspot and focus in engineering dielectric materials.

Alumina is commonly applied as a heat conduction material [17,18]. Due to the good thermal conductivity of alumina, in recent years, many scholars have conducted research on the effect of alumina on the dielectric properties of materials. Sudha *et al*. [19] reported on nanostructured amorphous alumina-modified polycarbonate composites, which showed the enhancement of dielectric constant over five times of pure polycarbonate and the loss decreased by 13.3% with 5 wt % Al_2_O_3_ as a filler and suggested that the composites could serve as a better electrical insulator in capacitors, printed circuit boards (PCBs) and electronic packaging industries [20]. Wang *et al*. [21] prepared a composite of Al_2_O_3_/LDPE (low density polyethylene) and showed that nano-Al_2_O_3_ could increase the electrical strength and electrical resistance of LDPE partly. It has been proved that Al_2_O_3_ as a functional filler can enhance the dielectric properties of inner insulation materials. It was found that with Al_2_O_3_ loading in composite materials, the relative permittivity increases, while the dielectric loss decreases; moreover, the breakdown strength will reduce accordingly [22,23]. However, nanoparticles tend to agglomerate in the polymer, which leads to increased dielectric loss and lower breakdown strength of the composites. Besides, at present, most investigators have focused on the spherical nano-alumina particles, and few have studied micro-Al_2_O_3_ particles. Moreover, the effects of Al_2_O_3_ particle shapes on dielectric properties of the composites were rarely reported.

Currently, a great majority of the polymer-based dielectric composites are formed by hot-pressing. This method is complicated, energy-intensive and requires a large amount of organic solvents, which is unfriendly to the environment [24,25]. Furthermore, evaporating the solvent during the curing process will make the surface of materials rough. In this work, the UV-curing technology was adopted to fabricate composite materials, due to the UV-curing technology being a high-efficiency and energy-saving, no evaporative volatile solvents and high-quality material surface treatment method that has been widely used in printing, electronic packaging and aerospace, etc. [26,27]. With the advancement of technology, a Japanese scholar has combined photocuring and 3D printing to print the sheet samples of alumina/UV-cured acrylic composites [23], which confirm the feasibility of UV-curing technology. Epoxy acrylic is the photocuring oligomer with the fast curing rate, which has excellent chemical resistance, good thermal stability and electrical properties. Epoxy acrylic is also the most widely used resin on the market [28–31]. Based on this, in this paper, the bisphenol A epoxy acrylic, diluent and photoinitiator were mixed in proper proportions to form the polymer matrix, two shapes of alumina particles sourced commercially were incorporated to enhance the relative permittivity of polymer materials, and cured by exposure to UV light. The dispersity of alumina particles in the matrix was observed by scanning electron microscopy (SEM), and the effect of alumina shapes on the dielectric properties of composites was investigated.

## 2. Experimental

### 2.1. Materials

Bisphenol A epoxy acrylic (EA) was provided by Nanjing Jiazhong Chemical Technology, China. Hexanediol diacrylate (HDDA, 99%) was used as a diluent to adjust the viscosity of the system, which was supplied by Henan Tianfu Chemical and used as received. A photoinitiator named 1-hydroxycycloethyl phenyl ketone (UV184, 98%) was purchased from Alfa Aesar and used as received. Irregular alumina (*i*-Al_2_O_3_) with a particle size of about 10–18 µm was provided by Taian Shengyuan Powder, China. Spherical alumina (*s*-Al_2_O_3_) with an average particle size of 15 µm was obtained from Ya'an Bestry Advanced Materials Co., Ltd, China.

### 2.2. Preparation for the Al_2_O_3_/EA composites

**Figure 1.**
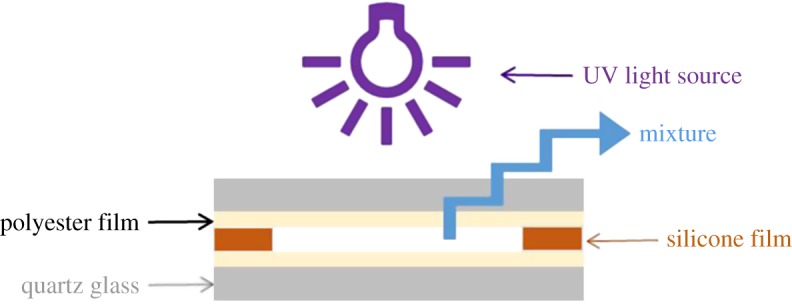
Diagrammatic sketch of UV-cured composites.

The Al_2_O_3_/EA composites were prepared by UV-curing. The specific preparation process is as follows: to form a matrix, bisphenol A epoxy acrylic (EA), HDDA and UV184 were mixed at a mass ratio of 20 : 4 : 1. Then, the irregular or spherical alumina was added to a polymer matrix as a filler with different volume content (10%, 20%, 30%, 40% and 50%). After mechanical stirring for 1 h, the homogeneous mixture was placed in a vacuum to remove air bubbles for 30 min at room temperature. Finally, the mixture was poured into the mould, the sheet samples with an average thickness of 0.2 mm were prepared by UV light irradiating the mixture for 20 s and the diagrammatic sketch of UV light curing is shown in figure 1. An unfilled resin sample was prepared as a control sample according to the above procedure.

### 2.3. Characterizations

To confirm whether the Al_2_O_3_/EA composites were cured completely, the structures of the composites were investigated by Fourier transform infrared spectroscopy (FTIR, Nicolet Impact-400, USA). The dielectric properties of the composite materials were dominated by the interfacial bonding between the matrix and the filler, and the homogeneous dispersity of filler particles in the matrix. The dispersion of the Al_2_O_3_ particles in the matrix and the interface state of the two phases were observed by a scanning electron microscope (SEM, SEC4500M, Korea) at a voltage of 10 kV, and samples were sprayed gold before testing. The dielectric properties of the composites were analysed by measuring the capacitance and dielectric loss tangent of the samples with an impedance analyser (Agilent E4980A, USA) from 50 Hz to 1 MHz under the measuring voltage of 1.0 V. A layer of conductive silver paste was placed on both sides of the sample before testing. The direct-current breakdown strength of composites was estimated on an LCR meter (YD-5750, Xinyuan Electric, China) with the voltage rise rate of 0.4 kV s^−1^. The test system for direct-current breakdown strength adopts ball electrodes with a diameter of 25 mm, which were placed in the transformer oil at 33°C to prevent surface flashover during the test.

## 3. Results and discussion

### 3.1. FTIR analysis

**Figure 2.**
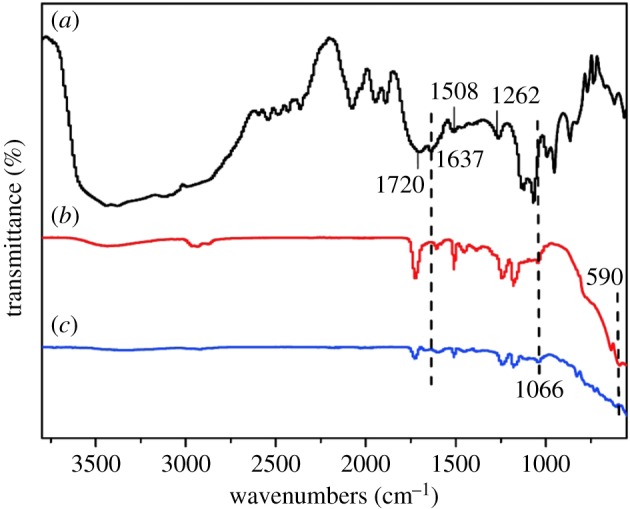
FTIR spectra of (*a*) uncured EA and cured composites, (*b*) *i*-Al_2_O_3_/EA and (*c*) *s*-Al_2_O_3_/EA.

The chemical structures of the uncured EA and the cured Al_2_O_3_/EA composites were characterized by FTIR spectroscopy, as depicted in figure 2. In the spectra of uncured resin, it can be seen that the absorption peak of C=O bonds was at 1720 cm^−1^, the characteristic peak of C=C bonds in epoxy acrylic oligomers was at 1637 cm^−1^, the skeletal vibration of the para-substituted benzene ring was at 1508 cm^−1^ and the C–O–C stretching vibration of fat aromatic ether was at 1262 cm^−1^. In addition, there are no characteristic peaks at 1066 and 590 cm^−1^ in the uncured resin, which are the bending vibrations of the OH and Al–O bonds, respectively, presented in Al_2_O_3_/EA composites. According to the UV-curing mechanism, under the effect of photoinitiator, the C=C bonds in functional monomers participate in the reaction to form a cross-linked network structure under the UV irradiation [27]. Obviously, there was not an absorption peak of C=C bonds in the cured samples. In other words, there was no acrylic oligomer in cured Al_2_O_3_/EA composites, which indicates that the Al_2_O_3_/EA composites have been fabricated successfully.

### 3.2. Microstructure of composites

**Figure 3.**
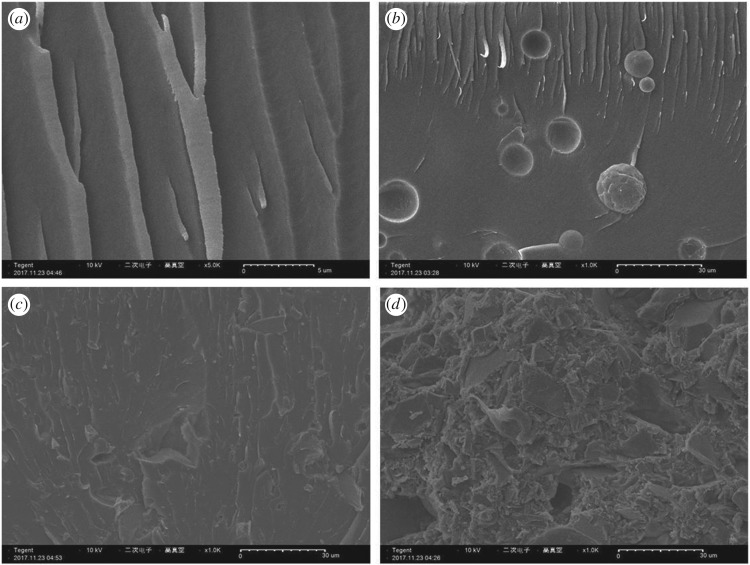
SEM of samples: (*a*) unfilled resin, (*b*) 10 vol% *s*-Al_2_O_3_, (*c*) 10 vol% *i*-Al_2_O_3_ and (*d*) 50 vol% *i*-Al_2_O_3_.

Figure 3 shows the SEM images of unfilled resin and Al_2_O_3_/EA composite samples. As seen from figure 3*b*, evidently non-uniform dispersion and deposition of *s*-Al_2_O_3_ particles can be observed in the resin, and almost a pure resin layer in the upper layer of the composite sample at the filler volume fraction of 10%, which is attributed to the fact that the *s*-Al_2_O_3_ particles have stronger flowability in the resin than the irregular ones, especially less filler content resulting in low viscosity of the system. Meanwhile, in figure 3*c*, the *i*-Al_2_O_3_ particles bond well to the matrix in the sample with *i*-Al_2_O_3_ of 10 vol% and present a homogeneous dispersity in the matrix. Significantly, increasing defects appeared as the filler loading increased, which might be because the interface is increased of Al_2_O_3_ particles and the air bubbles are hard to remove completely in the mixture with a high viscosity.

### 3.3. Dielectric properties

**Figure 4.**
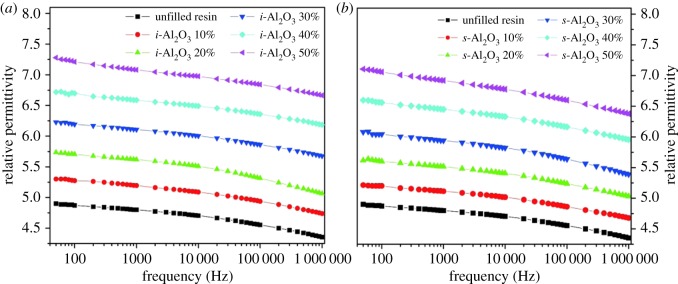
Frequency dependence of relative permittivity of composites: (*a*) *i*-Al_2_O_3_/EA and (*b*) *s*-Al_2_O_3_/EA with varied content measured at room temperature.

To characterize the dielectric properties of materials, the capacitance and dielectric loss tangent were measured. According to the formula about capacitance and relative permittivity, the frequency dependence of relative permittivity of all prepared samples at room temperature is shown in figure 4. It can be observed that the relative permittivity of all samples decreases with increasing frequency, which is attributed to the dielectric relaxation phenomena of materials. The permittivity of composites was dominated by the interfacial polarization. With the frequency increasing, the interfacial polarization cannot keep up with the change of frequency, resulting in the relative permittivity decreasing correspondingly [32]. Moreover, the curves illustrated that by increasing the content of Al_2_O_3_, the relative permittivity increased as expected and was all higher than that of pure resin [23,33–36]. Notably, the *i*-Al_2_O_3_/EA composites show higher relative permittivity compared with *s*-Al_2_O_3_/EA composites at the same Al_2_O_3_ concentration. It can be seen that the relative permittivity for *i*-Al_2_O_3_/EA composites increases from 5.30 to 7.28 as the volume fraction of filler incorporation is increased from 10% to 50% and 5.21 to 7.10 for *s*-Al_2_O_3_/EA at 50 Hz; however, the relative permittivity for unfilled resin is only 4.90.

**Figure 5.**
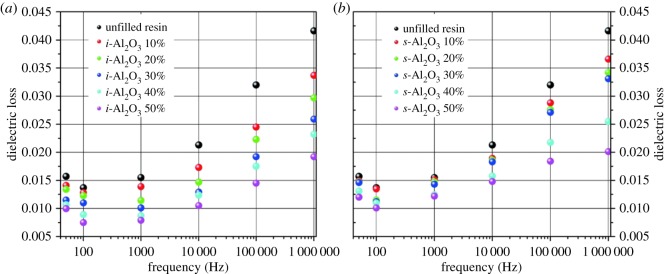
Frequency dependence of dielectric loss of composites: (*a*) *i*-Al_2_O_3_/EA and (*b*) *s*-Al_2_O_3_/EA with varied content measured at room temperature.

Figure 5 is a graph of dielectric loss of the resulted samples. As the dielectric loss of Al_2_O_3_ is lower than that of the resin [23], decreased dielectric loss was obtained as Al_2_O_3_ content increased. As seen from figure 5, the *i*-Al_2_O_3_/EA composites present lower dielectric loss compared with that of *s*-Al_2_O_3_/EA composites at the same content. The dielectric loss of *i*-Al_2_O_3_/EA is decreased from 0.0157 to 0.0099 and the dielectric loss of *s*-Al_2_O_3_/EA from 0.0157 to 0.012, which is mainly attributed to more defects that appeared in samples with *s*-Al_2_O_3_ resulting in a relatively higher loss.

Obviously, *i*-Al_2_O_3_/EA composites show better dielectric properties than the *s*-Al_2_O_3_/EA ones, which is due to the fact that spherical particles were prone to aggregate and deposit, resulting in more defects formed between the filler and matrix.

**Figure 6.**
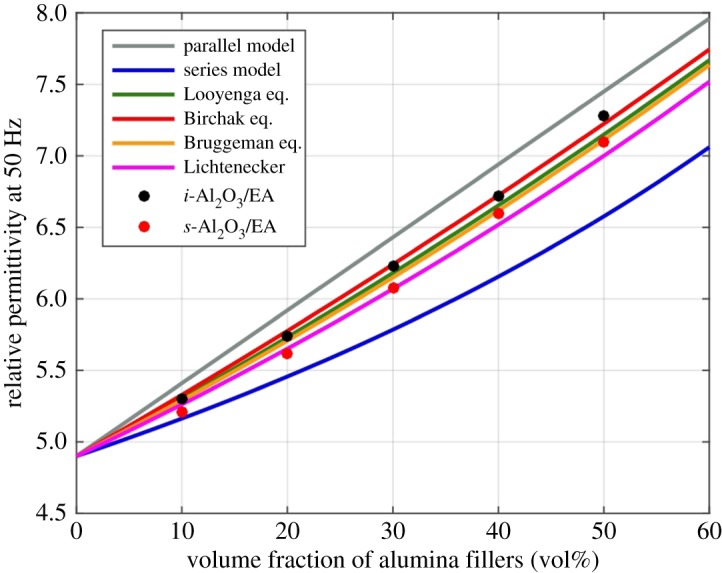
Dependence of relative permittivity on the filler volume fraction in composites at 50 Hz.

Figure 6 shows the dependence of relative permittivity on the volume fraction of alumina at 50 Hz. To verify the rationality of the experimental data, we compared a series of theoretical models [37]. In the figure, the experimental data of all the prepared materials are between the permittivity values calculated by the parallel capacitor model and the series capacitor model, which correspond to the maximum and minimum values of two media composites. Besides, the experimental data are accordant to the Bruggeman's model and the Lichtenecker–logarithmic model. Furthermore, to study the effect of filler shape on the permittivity of two media composites and better fit the experimental data, we introduced a power law model [38,39]. A power low model is often used in dielectric modelling of the composite system, and the effective permittivity of a two-component composite can be modelled by the following equation:

3.1 $${\varepsilon_{\mathrm{eff}}}^{\beta_{1}}=\phi_{1}{\varepsilon_{1}}^{\beta_{1}}+\;({1-\phi }_{1}){\varepsilon_{2}}^{\beta_{1}},$$

where *ɛ*_eff_, *ɛ*_1_ and *ɛ*_2_ are the relative permittivity of the composite material, the filler and the matrix, respectively, *Φ*_1_ is the volume fraction of the filler and *β*_1_ is the parameter of the shape and orientation of the filler within the composite. Here, the interface volume is not considered because the filler has micrometre-sized particles and its filling fraction is high. Looyenga [40] defines that *β*_1_ value is 1/3 for the spherical filler particles. We can see that the relative permittivity of the *s*-Al_2_O_3_/EA composite is closer to the Looyenga formula theoretical value. However, because of the incorporation of *i*-Al_2_O_3_, the relative permittivity tends to be in accordance with the Birchak formula [41], where *β*_1_ value is 1/2. The results show that the shape of the filler in composites has an important effect on the effective relative permittivity, and the larger the *β*_1_ value, the higher the effective relative permittivity of the composite [39].

### 3.4. Breakdown strength

Breakdown strength is a critical parameter for evaluating the properties of dielectric composite materials. According to Weibull distribution (IEEE Standard 930-2004), data were processed; *α* and *β*_2_ can be calculated by the following equations:

3.2 $$P_{i}=1-\exp\left[-{\left(\frac{E}{\alpha }\right)}^{\beta_{2}}\right],$$

where *E*, *α* and *β*_2_ are the experimental value, the characteristic breakdown strength and the shape parameter about filler dispersion, respectively. *P_i_* is the cumulative probability of dielectric breakdown, which should be calculated by the following formula as the number of test samples is less than 25:

3.3 $$P_{i}=\frac{i-0.44}{n+0.25},$$

**Figure 7.**
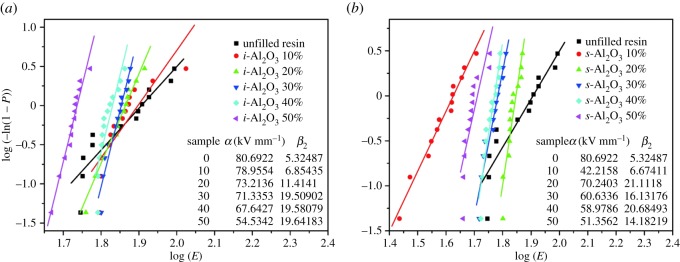
Two-parameter Weibull distribution of breakdown strength for the unfilled resin and composites with varied Al_2_O_3_ concentration: (*a*) *i*-Al_2_O_3_/EA and (*b*) *s*-Al_2_O_3_/EA.

The values of *α* and *β*_2_ can be obtained after the linear fitting, as shown in figure 7. The introduction of Al_2_O_3_ particles into the resin reduced the *α* value of EA [42], which is caused by interfacial defects and air bubbles. It can be seen from figure 3, with increasing alumina content, more defects are found inside the materials, which results in the increased charge accumulation and partial discharge inside the materials. Comparatively, the *i*-Al_2_O_3_/EA composites show relatively higher *α* than that of *s*-Al_2_O_3_/EA composites, because the spherical particles are easier to aggregate and more defects are found inside the composites. Besides, the shape parameter *β*_2_ of all Al_2_O_3_/EA composites is higher than that of unfilled resin, which reveals that the introduction of Al_2_O_3_ particles changes the breakdown mechanisms of EA. However, *α-*value decreased greatly at the filling amount of 10 vol%, which is a result of heavy defects in the sample.

## 4. Conclusion

Composite materials were prepared by UV-curing successfully, which is handy and energy-saving. The microstructure of composites illustrated that *i*-Al_2_O_3_ particles disperse homogeneously and the *s*-Al_2_O_3_ particles tend to aggregate and deposit in the resin. With the increasing alumina content, the relative permittivity of Al_2_O_3_/EA composites increased, the dielectric loss decreased accordingly. However, the incorporation of *i*-Al_2_O_3_ particles presents better dielectric properties when compared with *s*-Al_2_O_3_/EA. The relative permittivity of *i*-Al_2_O_3_/EA composites with the filler volume fraction of 50% is up to 7.28 and the dielectric loss is only 0.099 at 50 Hz. The comparison of experimental data with the models of permittivity of two media composite indicates that the experimental data are reasonable. Based on the power law model, the parameter of the shape of the *i*-Al_2_O_3_ in the *i*-Al_2_O_3_/EA composite is 1/2, which is higher than that of *s*-Al_2_O_3_ in the *s*-Al_2_O_3_/EA composites, indicating that *i*-Al_2_O_3_ has a more important effect on the permittivity of the composite than that of *s*-Al_2_O_3_. Moreover, the *i*-Al_2_O_3_/EA composites exhibit better breakdown performance than the *s*-Al_2_O_3_/EA composites. Such UV-curing moulding method is expected to be used in preparing more insulation materials of excellent performance combined with 3D printers.

## Supplementary Material

Reviewer comments

## Data accessibility

All data and research materials supporting the results are in the article.

## References

[1] Wang Y, Zhou X, Chen Q, Chu B, Zhang Q 2010 Recent development of high energy density polymers for dielectric capacitors. IEEE Trans. Dielectr. Electr. Insul. 17, 1036–1042. (10.1109/TDEI.2010.5539672)

[2] Barber P, Balasubramanian S, Anguchamy Y, Gong S, Wibowo A, Gao H, Ploehn HJ, zur Loye HC 2009 Polymer composite and nanocomposite dielectric materials for pulse power energy storage. Mater 2, 1697–1733. (10.3390/ma2041697)

[3] Wu S, Li W, Lin M, Burlingame Q, Chen Q, Payzant A, Xiao K, Zhang QM 2013 Aromatic polythiourea dielectrics with ultrahigh breakdown field strength, low dielectric loss, and high electric energy density. Adv. Mater. 25, 1734–1738. (10.1002/adma.201204072) 23315675

[4] Immergut EH, Brandrup J 1974 Polymer handbook, 2nd edn New York, NY: Wiley-Interscience.

[5] Zhu X, Zhu J, Zhou S, Liu Z, Ming N, Chan HLW, Choy CL, Wong KH, Hesse D 2005 Microstructure and dielectric properties of compositionally-graded (Ba_1−*x*_Sr_*x*_)TiO_3_ thin films. Mater. Sci. Eng. B 118, 219–224. (10.1016/j.mseb.2004.12.033)

[6] Ge PZ, Tang XG, Liu QX, Jiang YP, Li WH, Li B 2018 Temperature-dependent dielectric relaxation and high tunability of (Ba_1−*x*_Sr_*x*_)TiO**_3_** ceramics. J. Alloy Compd. 731, 70–77. (10.1016/j.jallcom.2017.09.330)

[7] Lombardi M, Guerriero A, Kortaberria G, Mondragon I, Sangermano M, Montanaro L 2011 Effect of the ceramic filler features on the properties of photopolymerized BaTiO**_3_**-acrylic composites. Polym. Composite 32, 1304–1312. (10.1002/pc.21154)

[8] Xing J, Radovic M, Muliana AH 2018 Elastic and dielectric properties of active Ag/BaTiO**_3_** composites. Exp. Mech. 58, 645–660. (10.1007/s11340-017-0271-5)

[9] Palade S, Pantazi A, Vulpe S, Berbecaru C, Tucureanu V, Oprea O, Negrea RF, Dragoman D 2017 Tunable dielectric properties in polyacrylonitrile/multiwall carbon nanotube composites. Polym. Composite 38, 1741–1748. (10.1002/pc.23744)

[10] Aaditya VB, Bharathesh BM, Harshitha R, Chaluvaraju BV, Raghavendra UP, Murugendrappa MV 2018 Study of dielectric properties of polypyrrole/titanium dioxide and polypyrrole/titanium dioxide-MWCNT nano composites. J. Mater. Sci. Mater. Electr. 29, 2848–2859. (10.1007/s10854-017-8214-6)

[11] Ghosh B, Tamayo Calderón RM, Espinoza-González R, Hevia SA 2017 Enhanced dielectric properties of PVDF/CaCu_3_Ti_4_O_12_:Ag composite films. Mater. Chem. Phys. 196, 302–309. (10.1016/j.matchemphys.2017.05.009)

[12] He D, Wang Y, Chen X, Deng Y 2017 Core–shell structured BaTiO**_3_**@Al**_2_**O**_3_** nanoparticles in polymer composites for dielectric loss suppression and breakdown strength enhancement. Compos. Part A Appl. S. 93, 137–143. (10.1016/j.compositesa.2016.11.025)

[13] Sinha D, Pillai PKC 1989 Ceramic-polymer composites as potential capacitor material. J. Mater. Sci. Lett. 8, 673–674. (10.1007/bf01730438)

[14] Paine RT, Pruss EA, Wood GL, Schwierkowski C, Hill RF, Chapelle C, Kroenke WJ 2001 Boron nitride fillers for organic polymer composites. ACS Symp. Ser. 804, 27–38. (10.1021/bk-2002-0804.ch004)

[15] Zhang X, Shen Y, Zhang Q, Gu L, Hu Y, Du J, Lin Y, Nan CW. 2015 Ultrahigh energy density of polymer nanocomposites containing BaTiO**_3_**@TiO**_2_** nanofibers by atomic-scale interface engineering. Adv. Mater. 27, 819–824. (10.1002/adma.201404101) 25492492

[16] Kim P, Jones SC, Hotchkiss PJ, Haddock JN, Kippelen B, Marder SR, Perry JW 2007 Phosphonic acid-modified barium titanate polymer nanocomposites with high permittivity and dielectric strength. Adv. Mater. 19, 1001–1005. (10.1002/adma.200602422)

[17] Tavman IH 1996 Thermal and mechanical properties of aluminum powder-filled high-density polyethylene composites. J. Appl. Polym. Sci. 62, 2161–2167. (10.1002/(SICI)1097-4628(19961219)62:12<2161::AID-APP19>3.0.CO;2-8)

[18] Wu X, Wang Y, Xie L, Yu J, Liu F, Jiang P 2013 Thermal and electrical properties of epoxy composites at high alumina loadings and various temperatures. Iran. Polym. J. 22, 61–73. (10.1007/s13726-012-0104-4)

[19] Sudha LK, Roy S, Rao KU 2017 Effect of nanofillers on the dielectric properties of nanostructured amorphous alumina modified polycarbonate composites. Mater. Today Proceed. 4, 9561–9565. (10.1016/j.matpr.2017.06.224)

[20] Tanaka T, Montanari GC, Mülhaupt R 2004 Polymer nanocomposites as dielectrics and electrical insulation-perspectives for processing technologies, material characterization and future applications. IEEE Trans. Dielectr. Electr. Insul. 11, 763–784. (10.1109/TDEI.2004.1349782)

[21] Wang W, Li S, Lin J, Min D 2016 Thermomechanical performances of polyethylene/alumina nanodielectrics by molecular motion in interface. IEEE Int. Confer. Dielectr. 1, 116–119. (10.1109/ICD.2016.7547557)

[22] Cho SD, Lee JY, Hyun JG, Paik KW 2004 Study on epoxy/BaTiO**_3_** composite embedded capacitor films (ECFs) for organic substrate applications. Mater. Sci. Eng. B 110, 233–239. (10.1016/j.mseb.2004.01.022)

[23] Kurimoto M, Ozaki H, Yamashita Y, Funabashi T, Kato T, Suzuoki Y 2016 Dielectric properties and 3D printing of UV-cured acrylic composite with alumina microfiller. IEEE Trans. Dielectr. Electr. Insul. 25, 2985–2992. (10.1109/TDEI.2016.005743)

[24] Alias A, Ahmad Z, Ismail AB 2011 Preparation of polyimide/Al**_2_**O**_3_** composite films as improved solid dielectrics. Mater. Sci. Eng. B 176, 799–804. (10.1016/j.mseb.2011.04.001)

[25] Yao M, You S, Peng Y 2017 Dielectric constant and energy density of poly(vinylidene fluoride) nanocomposites filled with core-shell structured BaTiO**_3_**@Al**_2_**O**_3_** nanoparticles. Ceram. Int. 43, 3127–3132. (10.1016/j.ceramint.2016.11.128)

[26] Xing W, Jie G, Song L, Wang X, Lv X, Hu Y 2011 Flame retardancy and thermal properties of epoxy acrylate resin/alpha-zirconium phosphate nanocomposites used for UV-curing flame retardant films. Mater. Chem. Phys. 125, 196–201. (10.1016/j.matchemphys.2010.09.006)

[27] Park YJ, Lim DH, Kim HJ, Park DS, Sung IK 2009 UV- and thermal-curing behaviors of dual-curable adhesives based on epoxy acrylate oligomers. Int. J. Adhes. Adhes. 19, 710–717. (10.1016/j.ijadhadh.2009.02.001)

[28] Liu P, Gu A, Liang L, Guan Q, Yuan L 2012 Preparation and properties of novel high performance UV-curable epoxy acrylate/hyperbranched polysiloxane coatings. Prog. Org. Coat. 74, 142–150. (10.1016/j.porgcoat.2011.11.026)

[29] Liu M, Mao X, Zhu H, Lin A, Wang D 2013 Water and corrosion resistance of epoxy–acrylic–amine waterborne coatings: effects of resin molecular weight, polar group and hydrophobic segment. Corros. Sci. 75, 106–113. (10.1016/j.corsci.2013.05.020)

[30] Geurts J, Bouman J, Overbeek A 2008 New waterborne acrylic binders for zero VOC paints. J. Coat. Technol. Res. 5, 57–63. (10.1007/s11998-007-9036-x)

[31] Tillet G, Boutevin B, Ameduri B 2011 Chemical reactions of polymer crosslinking and post-crosslinking at room and medium temperature. Prog. Polym. Sci. 36, 191–217. (10.1016/j.progpolymsci.2010.08.003)

[32] Zhan JY, Tian GF, Wu ZP, Qi SL, Wu DZ 2014 Preparation of polyimide/BaTiO**_3_**/Ag nanocomposite films via in situ technique and study of their dielectric behavior. Chin. J. Polym. Sci. 32, 424–431. (10.1007/s10118-014-1413-1)

[33] Wang Z, Zhou W, Sui X, Dong L, Cai H, Zuo J, Liu X, Chen Q 2016 Dielectric studies of Al nanoparticle reinforced epoxy resin composites. Polym. Composite 39, 887–894 (10.1002/pc.24012)

[34] Murudkar VV, Gaonkar AA, Deshpande VD, Mhaske ST 2016 Comparison of dielectric properties of polydimethylsiloxane (PDMS) grafted polyacrylates/nano alumina and nano silica composites. AIP Conf. Proc. 1728, 020622 (10.1063/1.4946673)

[35] Rajesh S, Jantunen H, Letz M, Pichler-Willhelm S 2011 Low temperature sintering and dielectric properties of alumina-filled glass composites for LTCC applications. Int. J. Appl. Ceram. Technol. 9, 52–59. (10.1111/j.1744-7402.2011.02684.x)

[36] He S, Hu J, Zhang C, Wang J, Chen L, Bian X, Lin J, Du X. 2018 Performance improvement in nano-alumina filled silicone rubber composites by using vinyl tri-methoxysilane. Polym. Test. 67, 295–301. (10.1016/j.polymertesting.2018.03.023)

[37] Vargas-Bernal R, Herrera-Pérez G, Calixto-Olalde ME, Tecpoyotl-Torres M 2013 Analysis of DC electrical conductivity models of carbon nanotube-polymer composites with potential application to nanometric electronic devices. J. Electr. Comput. Eng. 2013, 1–14. (10.1155/2013/179538)

[38] Todd MG, Shi FG 2005 Complex permittivity of composite systems: a comprehensive interphase approach. IEEE Trans. Dielectr. Electr. Insul. 12, 601–611. (10.1109/TDEI.2005.1453466)

[39] Wang W, Li S 2018 A transition of interface characteristics in LDPE/Al_2_O_3_ nanocomposites by permittivity simulation. IEEE Trans. Dielectr. Electr. Insul. 25, 2–12. (10.1109/TDEI.2018.005779)

[40] Looyenga H 1965 Dielectric constants of heterogeneous mixtures. Physica A. 31, 401–406. (10.1016/0031-8914(65)90045-5)

[41] Birchak JR, Gardner CG, Hipp JE, Victor JM 1974 High dielectric constant microwave probes for sensing soil moisture. Proc. IEEE 62, 93–98. (10.1109/PROC.1974.9388)

[42] Wang SJ, Zha JW, Wu YH, Ren L, Dang ZM, Wu J 2015 Preparation, microstructure and properties of polyethylene/alumina nanocomposites for HVDC insulation. IEEE Trans. Dielectr. Electr. Insul. 22, 3350–3356. (10.1109/TDEI.2015.004903)

